# Assessing coastal zooplankton in the St. Lawrence estuary: spatio-temporal patterns of taxonomic and functional biodiversity

**DOI:** 10.1093/plankt/fbae073

**Published:** 2025-01-30

**Authors:** Mélanie Santo, Piero Calosi, Gesche Winkler

**Affiliations:** Institut des Sciences de la Mer, Université du Québec à Rimouski, Québec-Océan, 310 Allée des Ursulines, G5L3A1, Rimouski, Québec, Canada; Laboratoire de Physiologie Écologique et Évolutive Marine, Département de Biologie, Chimie et Géographie, Québec-Océan, Université du Québec à Rimouski, 300 Allée des Ursulines, G5L3A1, Rimouski, Québec, Canada; Institut des Sciences de la Mer, Université du Québec à Rimouski, Québec-Océan, 310 Allée des Ursulines, G5L3A1, Rimouski, Québec, Canada

**Keywords:** biodiversity, zooplankton, functional traits, hotspot, coastal area

## Abstract

Biodiversity assessment promotes information on the state of an ecosystem. Zooplankton, as a sentinel group at the basis of aquatic food webs, are, thus, an important component to monitor for ecosystem conservation and management. For the first time, we characterized biodiversity of coastal zooplankton along the shallow Northern shoreline of the lower St. Lawrence estuary (LSLE) using an integrated taxonomic and trait-based approach. For 3 years (2019–2021), in July and October, the zooplankton community and environmental parameters were sampled at < 35 m depth. Mesozooplankton were identified at the lowest possible taxonomic level and assigned functional traits. Community structure and diversities revealed high spatio-temporal variations among three different geographic sectors and between seasons, mainly driven by water temperature, Chlorophyll-*a* concentration and less by salinity. Hotspots of taxonomic and functional diversities occurred in different sectors in the same month, underlining the complementarity of the two approaches. Seasonal shifts in functional diversity hotspots highlight how environmental variability affects biodiversity beyond taxonomic metrics alone. The results of our study in the LSLE establish a first robust baseline to improve our understanding of zooplankton dynamics in the coastal LSLE, to allow future tracking of ongoing change due to the increase of anthropogenic activities and climate changes and to support future monitoring efforts.

## INTRODUCTION

According to the objectives of the *Convention on Biological Diversity* (UN, 1992 ), biological diversity is defined as “the variability among living organisms and the ecological complexes of which they are part,” including “diversity within species, between species and of ecosystems,” and its conservation is recommended (UN, 1992) to maintain stability and resilience of ecosystems (Johnson *et al*., 2011). For this reason, management strategies commonly use biodiversity information to evaluate the state of ecosystems (Chiba *et al*., 2018). Biodiversity includes different elements, such as specific diversity (taxonomic) and ecological diversity (functional), which are interlinked (Gaston and Spicer, 2004) and determine the overall intrinsic properties of an ecosystem. Consequently, it is necessary to assess each of these elements to significantly improve our understanding of how ecosystems work and, therefore conservation and management (Bremner, 2008; Martini *et al*., 2021). Taxonomic diversity is based on individuals according to their taxonomic hierarchy (Gaston and Spicer, 2004), and functional diversity is based on taxa functional traits (Pomerleau *et al*., 2015). Functional traits represent specific morphological, physiological, life history and behavioral characteristics that determine the individual interactions with the environment and define the role of a species within an ecosystem (Barnett *et al*., 2007).

Zooplankton have a pivotal position in food webs, linking primary producers to carnivorous predators, and are useful indicators to estimate ecosystem conditions and changes (Usov *et al*., 2019; Llope *et al*., 2020). Zooplankton species respond to the physical, chemical and biotic variables of their environment, such as temperature, salinity, food quantity and quality (Lindegren *et al*., 2020), which can affect their distribution limits (Laprise and Dodson, 1994; Helenius *et al*., 2017; Lindegren *et al*., 2020) and the degree of ecological niche overlap (Hutchinson and Edmondson, 1957). These ecophysiological responses in species to varying environmental conditions will be reflected by a specific species composition and by a combination of functional traits. Defining these combinations could help in understanding how environmental factors contribute to the structure of zooplankton communities and affect their functioning (Litchman *et al*., 2013). Moreover, monitoring the distribution patterns of zooplankton enables the detection of environmental changes (Russel, 1936; Beaugrand, 2004), as these organisms, which are mostly short-lived, respond rapidly and sensitively to changes in abiotic and biotic factors. Therefore, studying the dynamics of zooplankton communities is crucial for the conservation and management of marine ecosystems, particularly in protected areas and regions of ecological and economic significance (Johnson *et al*., 2011). One such area is the lower St. Lawrence estuary (LSLE) (QC, Canada), which has been designated as a key coastal zone within Canada's “Coastal Environmental Baseline Program” under the “Ocean Protection Plan”. Coastal zones serve as vital links between terrestrial and open marine waters, exposing them to numerous anthropogenic pressures (Dufour *et al*., 2010) and rendering them sensitive to environmental changes (Lu *et al*., 2018).

The LSLE (200 km long, 40 km wide and 350 m deep) shows high spatial and temporal variations in abiotic and biotic factors (Plourde *et al*., 2002). Its hydrodynamics are primarily controlled by the St. Lawrence River runoff, tides and ice cover influencing the physico-chemical factors, such as temperature and salinity throughout the seasons (Blais *et al*., 2018; Galbraith *et al*., 2022). The LSLE provides a habitat for key taxa, such as krill and copepods, as well as a sanctuary for emblematic marine mammals, such as the beluga and the blue whale (Dufour *et al*., 2010; Gavrilchuk *et al*., 2014; Savenkoff *et al*., 2017). In addition, commercial and recreational fisheries, as well as tourist activities (*e.g.* whale watching), occur in this region. Zooplankton supports this diverse ecosystem, which is mainly composed of lipid-rich and big copepod species, such as *Calanus* spp. in summer. The proportion of small species, such as *Oithona* spp., *Acartia* spp. and *Microcalanus* spp., increases in autumn (Plourde *et al*., 2002; Blais *et al*., 2023). However, the coastal zone of LSLE has been scarcely studied so far, indicating lower abundances of all species, particularly of *Calanus* spp. in the shallow area (30–50 m) compared to the deep central LSLE (> 50 m, Plourde *et al*., 2002). While most of the zooplankton studies of the LSLE have focused on deep pelagic areas (Runge and Simard, 1990; Plourde *et al*., 2002), there have been no studies conducted on coastal zooplankton communities along the shallow northern shoreline (< 30 m) of LSLE until now.

Therefore, the aim of our study was to evaluate the natural variability in coastal zooplankton community structure and diversity of the LSLE that is driven by environmental conditions. Using a combined approach of taxonomic and functional biodiversity, we characterized spatial and temporal variation in the structure, diversity and dynamics of zooplankton communities in relation to environmental conditions focusing on two different seasons, summer and autumn, to account for community succession, in three consecutive years from 2019 to 2021. These complementary approaches provide for the first time an improved, more holistic assessment of biodiversity of coastal zooplankton in the LSLE.

## MATERIAL AND METHODS

### Study area

**Fig. 1:**
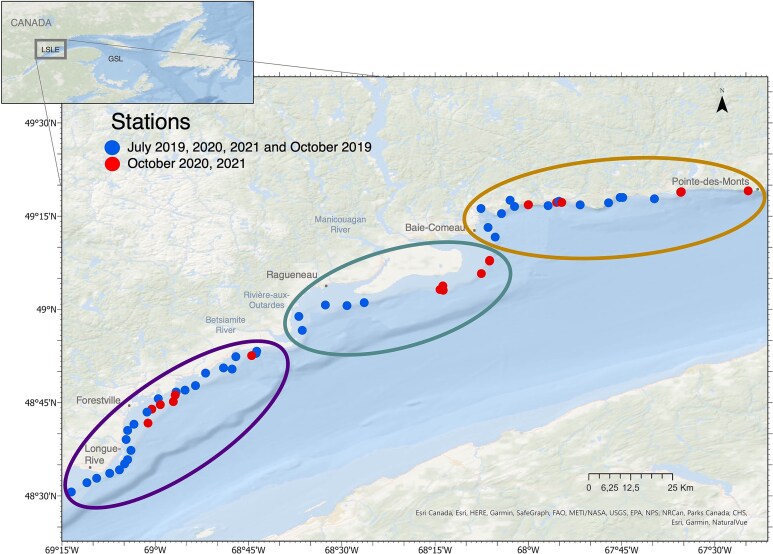
Map describing the sampling stations located along the north coast of LSLE. Stations in blue were sampled in July 2019, 2020 and 2021 and October 2019. Stations in red were sampled in October 2020 and 2021. Ellipsoids represent the different sectors: “Forestville” in purple (left), “Manicouagan” in green (middle) and “B-C” in orange (right). Global map on the top is the location of the study area in the St. Lawrence estuary. GSL, Gulf of St. Lawrence.

The study area was situated between 48.51°N, 69.22°W and 49.31°N, 67.41°W on the northern coast of LSLE (Fig. 1). Based on differences in contribution of water masses from tributary rivers, the St. Lawrence River and/or the Gulf of St. Lawrence (GSL) (Pinet *et al*., 2011b) and due to the geomorphological differences, this stretch of 170 km was separated in three sectors in this study, namely “Forestville”, “Manicouagan” and “Baie-Comeau (B-C)” (Fig. 1). Indeed, around Forestville, the substrate was characterized as fine sand and silt with a large tabular seafloor and a light slope, whereas around B-C, the coastal zone was constituted by sands with a rocky geomorphological signature and a steep slope close to the shoreline (Pinet *et al*., 2011a). The sector Manicouagan (near Ragueneau) was highly influenced by tributary rivers, such as the Betsiamite River, the Rivière-aux-Outardes and the Manicouagan River (El-Sabh, 1979; Pinet *et al*., 2011b) and was characterized by submarine fans of sand to silt, with a gently slope, low depth and a large tabular seafloor.

### Sampling design

Sampling campaigns were conducted in July and October 2019, 2020 and 2021. During each campaign, between 6 and 37 stations were sampled in the coastal zone at depths between 3 and 35 m and < 1 km from the coast, between Longue-Rive and Pointe-des-Monts (Fig. 1 and Table S1). Three research vessels were used depending on their availability (ISMER-UQAR MACOMA 7 m, CCGS LEIM and CCGS PERLEY 22 m, Table S1), combining the sampling effort from campaigns by the Institut des Sciences de la Mer (ISMER-UQAR) and the Maurice Lamontagne Institute (DFO). The variation in the number of stations over months was due to meteorological conditions that limited the sampling effort.

### Environmental variables

At each sampling station, vertical profiles of temperature, salinity, fluorescence, pH, dissolved oxygen and turbidity were obtained by a CTD probe (SBE19plus, Sea-Bird Scientific, Bellevue, WA, US). Seawater samples were collected with a 5 L-Niskin bottle at 1 m depth for nutrients (NO_2_ + NO_3_, PO_4_) and Chlorophyll *a* (Chl *a*) concentration analyses. For nutrient analyses, water samples (12 mL) were filtered using 0.2 μm cellulose acetate filters and stored at −20°C until analyses which were performed with an automated chemistry analyzer (AA500 Autoanalyzer, SEAL Analytical, Mequon, WI, US), according to the standard method of the JGOFS protocol (JGOFS, 1994). However, concentrations of nutrients were not available for all months, depending on vessel and sampling campaigns (see Table S1).

For Chl *a* analyses, water samples (100–300 mL) were filtered through Whatman GF/F filters (25 mm diameter). Pigments were directly extracted with 90% acetone during 20 h at 4°C and then Chl *a* concentrations were measured with a fluorometer (10-AU-005-CE, Turner-Design, San Jose, CA, US), according to the protocol by Mundy *et al*. (2011), and then used to calibrate the CTD fluorescence probe.

### Zooplankton

Mesozooplankton was collected with a 200 μm mesh standard plankton net (1 m diameter) in oblique hauls throughout the entire water column. The filtered volume (2–55 m^3^) was determined with a flowmeter (2030R mechanical Flowmeter, General Oceanic, Miami, FL, US) and used for abundance calculations. Samples were fixed and preserved in 95% ethanol.

Zooplankton specimens were identified to the lowest possible taxonomic level, staged and counted using a stereoscopic microscope (SZX2, Olympus, Tokyo, Japan—max. 11.5x10). For each sample, 1/100–1/5 fraction was analyzed with a Hensen-Stempel pipette of 5 mL to calculate abundance (ind. m^−3^), or at least 400 copepods (all stages) and 30 *Calanus* spp. were counted to obtain a better representation of the species. In total, the counting precision obtained at a 95% confidence level was 90%, according to Harris *et al*. (2000). Copepods were identified at the species level when possible, and other taxa at the genus or species level, based on identification keys according to the literature (Razouls *et al*., 2005-2022). Because we used a 200 μm mesh, nauplii of small copepods species were not retained, therefore all copepods nauplii were removed from further numerical analyses and only copepodite stages (CI to CV) and the adult stage (CVI) were considered here. All taxa and stages identified are shown in Table S2.

Biomass data expressed as carbon concentration (mg C m^−3^) were calculated based on sizes and according to specialized literature (Table S3). Four functional traits were chosen, as they represent each functional trait typology (according to Martini *et al*., 2021): life cycle, trophic type, feeding strategy and size, each of them at various levels (Table I and S2).

**Table I:** List of functional traits used to classify species functional diversity. Life cycle: taxa which spend all their lifetime within the plankton (holoplankton) or only part of their life cycle, such as larval stages (meroplankton); categories of Trophic type and Feeding strategy were determined according to Kiørboe (2011) and Brun *et al.*  (2017); Size: based on minimum and maximum of taxa adult size, CVI stage for copepod species and specific size of development for other taxa.

Life cycle	Holoplankton	
	Meroplankton	
Trophic type	Omnivore	
	Herbivore	
	Carnivore	
	Non-feeding	
	Detritivore	
Feeding strategy	Active ambush feeder	
	Passive ambush feeder	
	Cruise feeder	
	Current feeder	
	Mixed feeder	
Size	Class	(mm)
	1	[0.2; 0.5]
	2	[0.5; 1.0]
	3	[1.0; 1.5]
	4	[1.5; 2.5]
	5	[2.5; 5.0]
	6	[5.0; 10.0]
	7	>10.0

### Numerical analyses

Taxa abundances were square-root transformed to reduce the effect of dominant taxa (Clarke *et al*., 2014). Taxonomic diversity indices such as species richness (S), Shannon-Wiener index (H′) and Pielou evenness (J’) were calculated with the “vegan” R package (R Core Team, 2021, version 4.1.1). Three functional diversity indices were chosen: *functional richness* (FRic) that “measures the amount of niche space occupied by the species within a community”; *functional divergence* (FDiv) that “measures the abundance-weighted functional differences among species within a community”; and *functional evenness* (FEve) that describes “how evenly spread the species (or abundance) of a community are in the niche space” (Mason *et al*., 2008). These indices were chosen because they describe independent aspects of species distribution in the niche space (Mason *et al*., 2008), and they can be compared with their analogous taxonomic diversity indices. The functional indices were calculated according to Villéger *et al*. (2008) with “mFD” R package based on Gower distance.

Maps of biodiversity hotspots and coldspots (for both taxonomic and functional diversity indices) were compiled using Getis-Ord-Gi ® from ArcGIS® Pro software. Due to a low number of stations covered in some months (Table S1), hotspot and coldspot analyses were performed at the level of month (“July” and “October”) with the 3 years (2019–2021) pooled to ensure the robustness of the analyses, by including a minimum of 30 sampling stations.

In order to determine which taxa contributed the most to dissimilarity among sectors, months and years, a SIMPER routine (Primer-e® + 7 software) was used. In addition, non-parametric permutational multivariate analysis of variance (PERMANOVA), based on Euclidean distance, was used to test for differences of total zooplankton abundance and diversity indices among geographic sectors, months and years, applying a Bonferroni correction to the post-hoc test results (“vegan” package, R Core Team, 2021). To test for the presence of differences in zooplankton assemblage composition among sectors, months and years, PERMANOVA based on Bray–Curtis similarity distance was applied, followed by a Monte Carlo post-hoc test (Primer-e® + 7; Clarke *et al*., 2014).

Prior to performing correlation analyses of zooplankton abundances, diversity indices and composition with environmental parameters, collinearity of the latter was investigated with Draftman plots (“Ggally” R package) and principal component analyses. Only one or two collinear environmental variables *per* collinear group were kept. Turbidity and pH were found to be collinear with Chl *a* and temperature, respectively, and thus were removed from further analyses. Distance-based redundancy analysis (dbRDA) was used to characterize differences in the community among groups (year, month and sector) and to explore relationships with temperature, salinity and Chl *a* (Primer-e® + 7). Finally, to determine how environmental variables affected zooplankton abundance and diversity, generalized additive models (GAMs) were applied using the “mgcv” R package. The best model was determined depending on the lowest ∆AIC using the “dredge” function from R package “MuMIn” (Table S4), and the change points of each GAM model plot were determined with the “EnvCpt” R package (R Core Team, 2021).

## RESULTS

### Environmental conditions

**Fig. 2:**
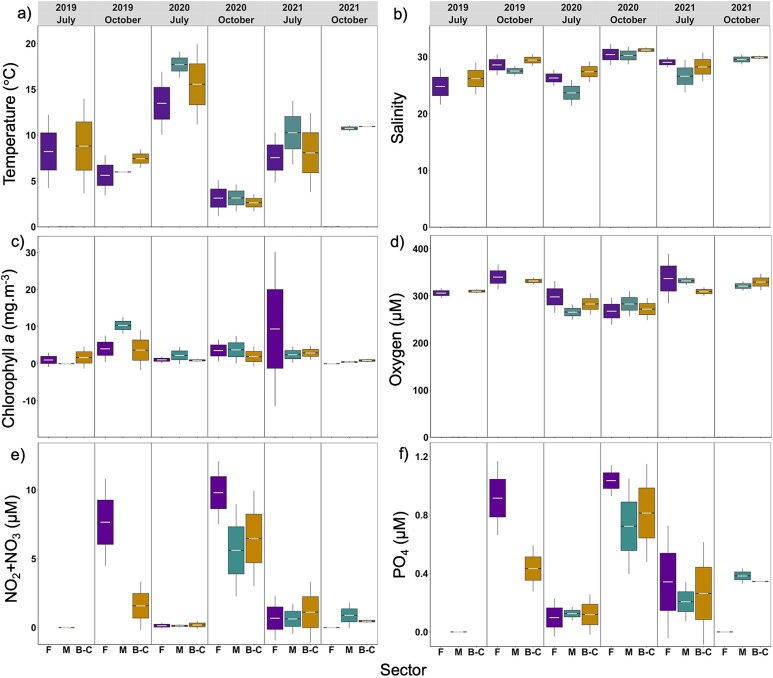
Mean seawater (**a**) temperature, (**b**) salinity, (**c**) Chl *a*, (**d**) oxygen, (**e**) NO_2_ + NO_3_ (nitrite + nitrate) and (**f**) PO_4_ (phosphate) values *per* sector, month and year. Forestville (F) is given in purple, Manicouagan (M) in green and B-C in yellow. Horizontal gray lines represent the mean. The top and bottom of the box represent + and—standard error respectively. The bars represent the 95% of the normal distribution. Please notice that y-axes have different scales and units.

Environmental conditions of the coastal zone of the LSLE varied strongly with season and differed among sectors, and this pattern was stable over the 3 years (Fig. 2, Table S5). Mean temperatures were higher in July than in October for all sectors, with a maximum of 17.70 ± 0.72°C in Manicouagan in July 2020 and a minimum of 2.62 ± 0.47°C in B-C in October 2020 (Table S5 and Table S6), except in 2021, when mean temperatures were similar in July and October. Mean salinity ranged between 23.67 ± 1.16 and 31.20 ± 0.24 and was higher in October compared to July (Table S5), except for 2021, when no significant difference was found between months (Fig. 2b). Similarly to salinity, mean Chl-*a* concentrations were higher in October compared to July (Table S6, Fig. 2c), except in 2021, when higher concentrations were found in July (Table S5 and Fig. 2c). Mean Chl-*a* concentrations ranged from 0.47 ± 0.12 mg m^−3^ (Manicouagan, October 2021) to 10.34 ± 1.12 mg m^−3^ (Manicouagan, October 2019). Oxygen concentrations were similar between months of the same year, ranging between 265.59 ± 8.07 (Manicouagan, July 2020) and 336.74 ± 26.73 μM (Forestville, July 2021) (Table S5 and Fig. 2d). Mean nutrient concentrations (NO_2_ + NO_3_ and PO_4_) were significantly higher in October than in July (Table S5). NO_2_ + NO_3_ concentrations ranged between 0.12 ± 0.05 and 1.11 ± 1.12 μM (Fig. 2e) and PO_4_ concentration between 0.09 ± 0.06 and 1.03 ± 0.04 μM (Fig. 2f).

In general, Forestville showed the lowest mean seawater temperatures in July and the highest mean nutrient concentrations in October among all sectors. Manicouagan was characterized by highest seawater temperature, especially in July (17.70 ± 0.72°C in 2020), by lower salinity and lower nutrients concentrations than the other sectors. B-C was the saltiest sector (except in July 2021), and mostly intermediate nutrients concentrations compared to the other sectors (Fig. 2).

### Taxonomic approach

#### Zooplankton community

**Fig. 3:**
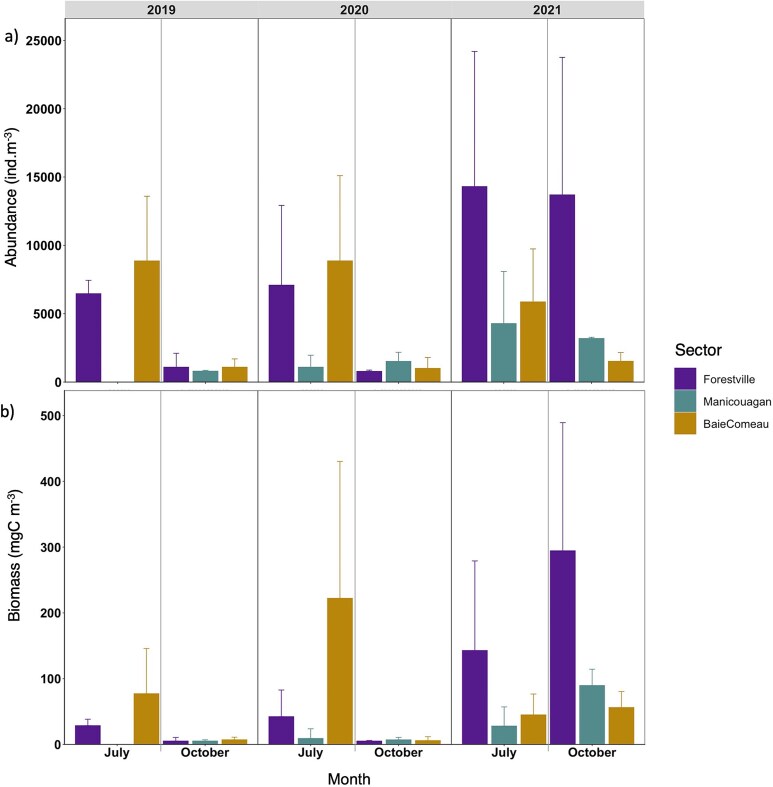
Means of (**a**) total abundance (ind. m^−3^) and of (**b**) total biomass (mgC m^−3^) *per* sector, month and year. Bars indicate standard error.

In 2019 and 2020, higher mean total zooplankton abundances were observed in July compared to October. For instance, in 2020, the mean abundance in July was 17 039 ± 5718 ind. m^−3^, while in October it was 3292 ± 1552 ind. m^−3^ (Table S7). In contrast, the abundances in July and October 2021 were more comparable, with 24 449 ± 17 569 ind. m^−3^ in July and 18 400 ± 10 797 ind. m^−3^ in October (Fig. 3a and Table S6). Spatial patterns in mean total abundances were similar in 2019 and 2020 but shifted in 2021. In July 2019 and 2020, there were significant differences in abundances among sectors, with high abundances observed in Forestville and B-C (>5000 ind. m^−3^), while Manicouagan consistently had lower abundances (<2000 ind. m^−3^) (Fig. 3a). In October of these years, all sectors exhibited similarly low abundances (<2000 ind. m^−3^), with the lowest mean total abundance of 612.7 ± 298.9 ind. m^−3^ recorded in Forestville. A notable shift occurred in 2021, with Forestville showing the highest mean total abundance of 14303.7 ± 9891.0 ind. m^−3^ in July (Fig. 3a). In both July and October 2021, zooplankton abundances in Forestville were three to five times higher than those in Manicouagan and B-C.

Similar temporal patterns were found for mean total biomass, with higher values in July (273.6 ± 263.1 mgC m^−3^ in 2020) than in October (18.0 ± 10.4 mgC m^−3^ in 2020), except in 2021 when the highest carbon concentration was found in October (440.1 ± 243.8 mgC m^−3^) (Fig. 3b). Spatial structure of biomass followed the same pattern of abundance, with higher biomass in B-C than Forestville and Manicouagan in 2019 and 2020, but in 2021, higher biomass was found in Forestville than B-C (Fig. 3b).

**Fig. 4:**
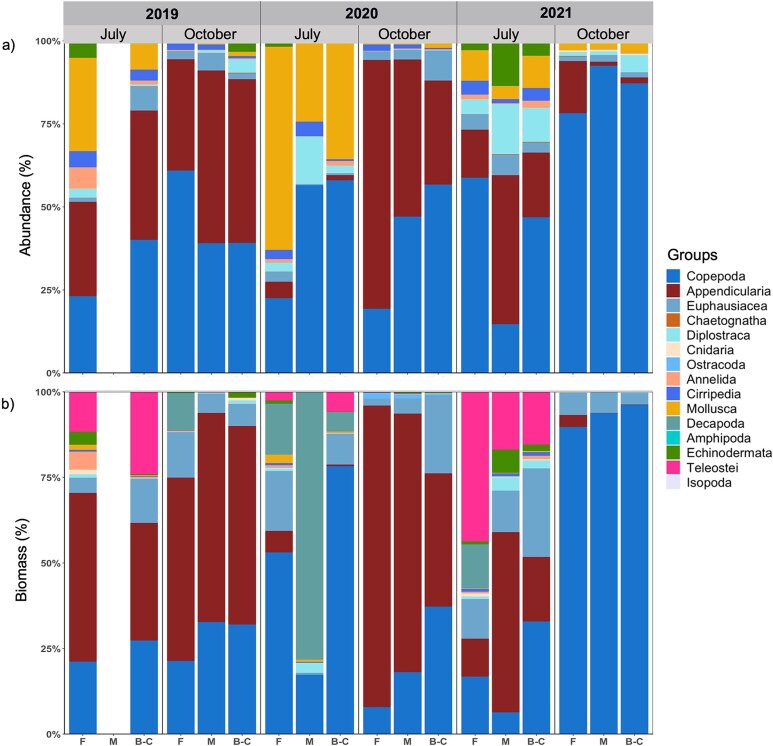
Zooplankton community structure expressed as proportion of (**a**) abundance (%) and (**b**) biomass (%) of taxonomic groups *per* sector (F, Forestville; M, Manicouagan; B-C, Baie-Comeau), month and year.

A total of 59 taxa were identified, spanning from the phylum (e.g. Chaetognatha) and class (e.g. Polychaeta) to the species level (Table S2). Species composition was highly variable and differed among years, months and sectors, as indicated by a significant interaction term of *year, months* and *sectors* (PERMANOVA, Table S8). The zooplankton community was mostly composed of Copepoda (30–80.7% throughout months and sectors, Fig. 4a). High proportions of Mollusca (49.7% overall, but especially in Forestville 60.1%) were found in July 2020 compared to the same month in the other years (8.9–19.8%). Appendicularia were dominant (40–43.8%) in October 2019 and 2020 but not in 2021 (12.5%). The contributions of Diplostraca were variable, ranging between 0.1 and 6.1%, with higher percentages in July 2020 and 2021 as well as in October 2021. Echinodermata showed a higher proportion in July than in October for all years (Fig. 4a).

The zooplankton composition, based on carbon biomass was more variable than based on abundances, particularly in July. In this month, the community was dominated by Appendicularia and Copepoda in 2019, by Copepoda and Decapoda in 2020 and by an increased contribution of Teleostei in 2021. Highest contribution of Appendicularia biomass was revealed in October 2019 and 2020, while in October 2021 Copepoda dominated (Fig. 4b).

**Fig. 5:**
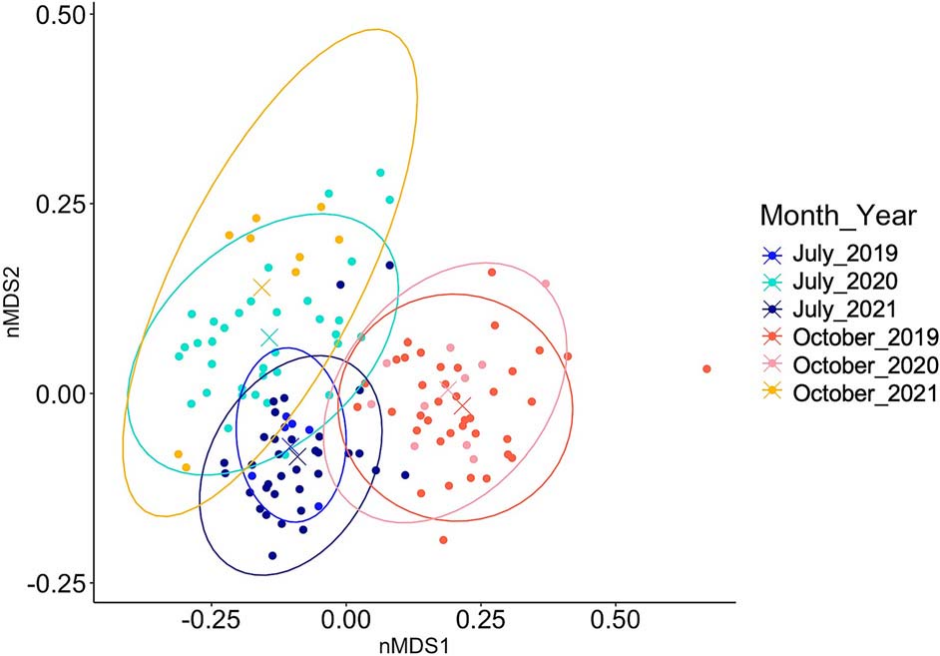
Non-metric multidimensional scaling based on species assemblage (Bray–Curtis dissimilarity) *per* month and year. The “X” in the middle of the ellipses represents the centroid of the groups. Stress = 0.13.

Temporal structure of the community indicated seasonality in each year. Comparing species assemblages based on the distance of centroid of Bray–Curtis dissimilarity at the level of month and year revealed a constant differentiation between July and October, with similar assemblages in July 2019 and 2021, as well as October 2019 and 2020 (Fig. 5 and Table S9). On the contrary, the zooplankton community in July 2020 and October 2021 were different from the ones in July and October of the other 2 years, respectively (Fig. 5 and Table S10).

Spatial structure of communities among sectors was found in July 2020 and 2021 and in October 2019 (Table S9). However, dissimilarities among sectors within the same month did not exceed 60% (Table S11). Five taxa mainly contributed to species composition (across the sectors), namely Bivalvia, Cirripedia, *Fritillaria* sp., *Acartia longiremis*, and *Oithona similis* (Table II). Their abundances were higher in July than in October (Table S7).

**Fig. 6:**
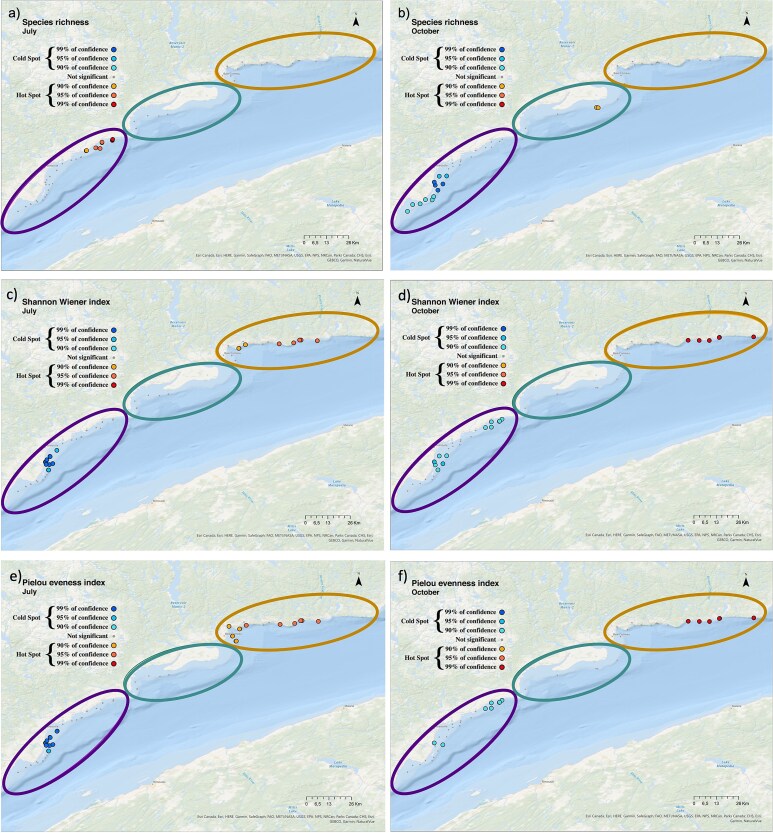
Maps indicating the distribution of hotspots (red shades) and coldspots (blue shades) of (**a, b**) species richness, (**c, d**) Shannon-Wiener index and (**e, f**) Pielou index in July (left panels) and October (right panels) all years pooled, in the sampling area. Sectors are represented by ellipsoids, “Forestville” in purple (left), “Manicouagan” in green (middle) and “B-C” in orange (right).

The spatial distribution of biodiversity hotspots and coldspots varied between the 2 months and among indices (species richness, Shannon-Wiener and Pielou) along the coastal zone. Hotspots in species richness (S) were found in the Forestville sector in July. In contrast, in October, coldspots were detected in Forestville and hotspots were found in Manicouagan (Fig. 6a and b). For both months, hotspots of Shannon-Wiener diversity and the Pielou evenness occurred in B-C, whereas Forestville showed coldspots for these diversity indices (Fig. 6c–f).

#### Community-environment interactions

**Fig. 7:**
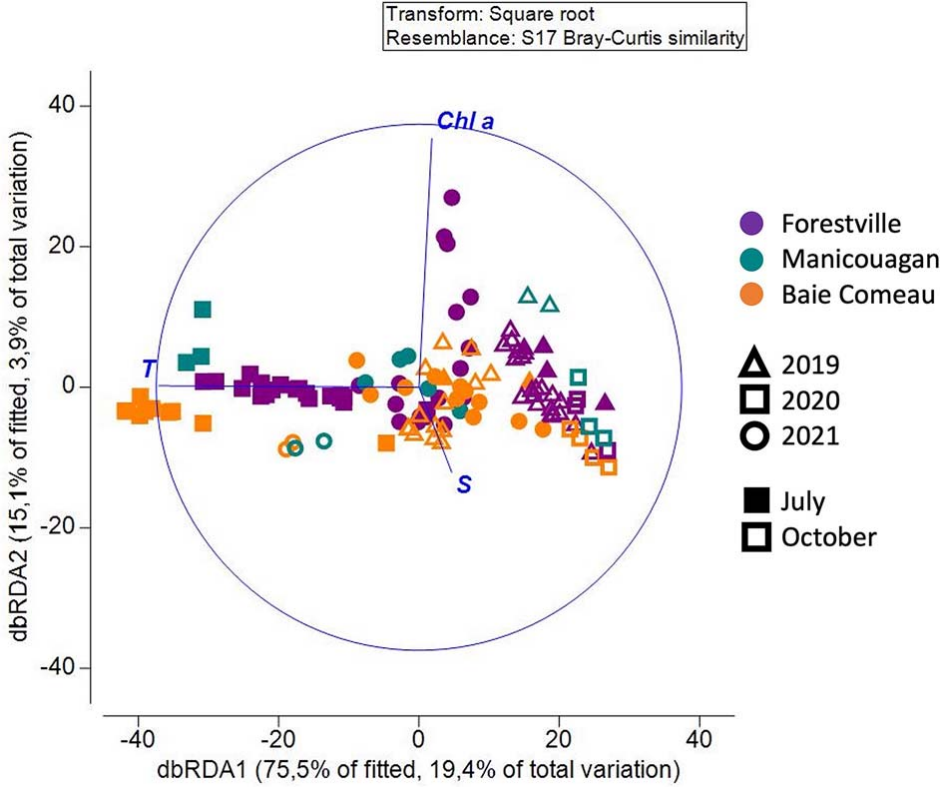
dbRDA of zooplankton community composition based on Bray–Curtis dissimilarity, correlated with three environmental parameters (temperature, salinity and Chl *a*). Colors represent sectors, symbols represent years, and full symbols correspond to July and empty symbols correspond to October.

**Fig. 8:**
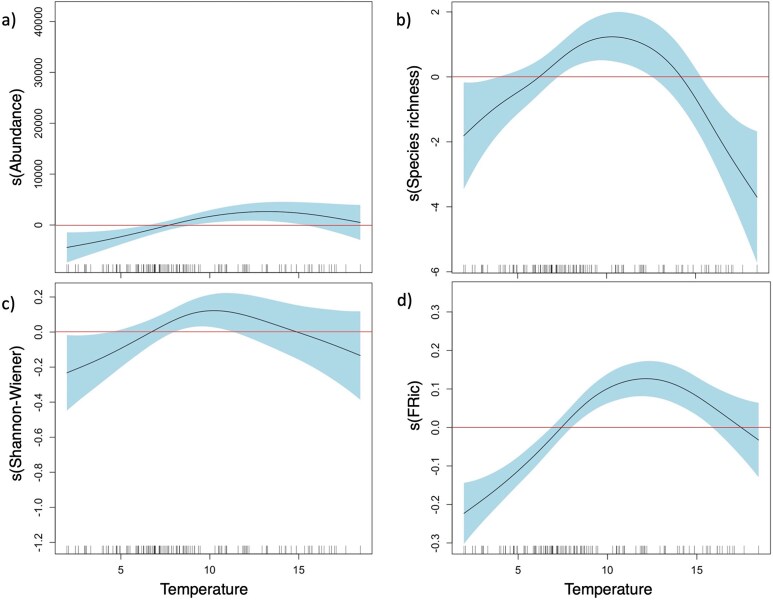
Smoothed plot representing the partial effects of temperature (°C) on (**a**) total mean abundance, (**b**) species richness, (**c**) Shannon-Wiener index and (**d**) FRic. Zone in blue represent the confidence interval of 95%. Positive effects are above zero (red line) of the contribution of the smoothed factor (vertical axis). Horizontal axis represented the temperature gradient.

The zooplankton community composition, based on Bray–Curtis dissimilarities, was influenced by different environmental variables (Fig. 7). Temperature emerged as the main driver (dbRDA1: 75.5% of fitted, 19.4% of total variation) that mostly explained the variability found in zooplankton composition. Particularly, in 2020, distinct zooplankton communities were correlated to high temperatures in July and low temperatures in October. In contrast, in October 2021, the community was mainly influenced by relatively warmer temperatures compared to October 2019 and 2020. However, the results of October 2021 should be interpreted with caution because only environmental data from four stations were available. Apart from temperature, the community composition was influenced by two secondary factors (dbRDA2: 15.1% of fitted, 3.9% of total variation), namely salinity and Chl *a,* strongly positively correlated with the communities in October (2019, 2020) and July (2021), respectively. Temperature and Chl *a* also had main influence on abundance and diversity indices. The threshold of a positive temperature effect on total abundance was at 7.5°C, based on GAM analysis (Fig. 8a; Table S12). A positive effect of temperature was similar on species richness and Shannon-Wiener index, ranging between 6.4–14.2°C and 6.1–13.6°C, respectively (Fig. 8b and c). Below and above these temperature ranges, abundance and diversity indices were negatively affected by temperature (Table S12). Total abundance was positively correlated with Chl *a,* whereas the Shannon-Wiener index and the Pielou evenness were negatively correlated with Chl *a* (Table S12). Predictability of the relationship was high for abundance (deviance explained: 42.8%) but low for all taxonomic diversity indices (deviance explained: 12.6–18.4%, Table S12).

### Functional approach

#### Functional composition and diversities

**Fig. 9:**
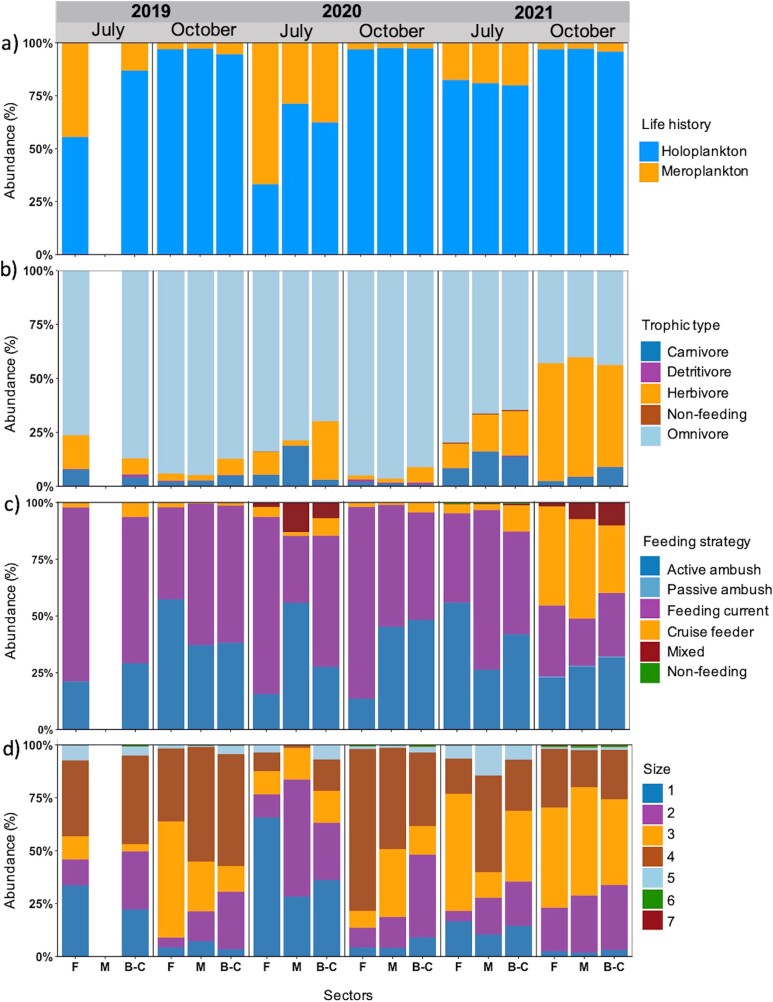
Composition of functional traits (%) based on abundance data for (**a**) life history, (**b**) trophic type, (**c**) feeding strategy and (**d**) size class of the zooplankton communities *per* sector (F, Forestville; M, Manicouagan; B-C, Baie-Comeau), month and year.

The zooplankton communities were mostly composed of holoplankton, independently of months or years (30–90%). The proportion of meroplankton was higher in July than in October, especially in 2020 (30–70%) (Table S13; Fig. 9a). Omnivores dominated in abundance in both months (45–90%). Herbivores occurred in higher proportion in July compared to October, except for October 2021 when they showed the highest overall proportion. Carnivores were present in larger proportions in July (10–20%) (Fig. 9b). In general, feeding current and active ambush strategies were proportionally more abundant than the four other feeding strategies. Mixed feeding was only present in July 2020 and October 2021 and was absent in the other months. Cruise feeders were mostly present in July, except in October 2021, when they occurred in highest overall proportion (Fig. 9c). The majority of zooplankton (≥50%) was represented in size classes 2 to 4 (0.5–2.5 mm). The smallest size class 1 (0.2–0.5 mm) and the medium size class 5 (2.5–5.0 mm) showed higher contribution in July than in October, while the proportion of largest size classes 6 and 7 (>5.0 mm) was very small and concentrated in October (Fig. 9d).

**Fig. 10:**
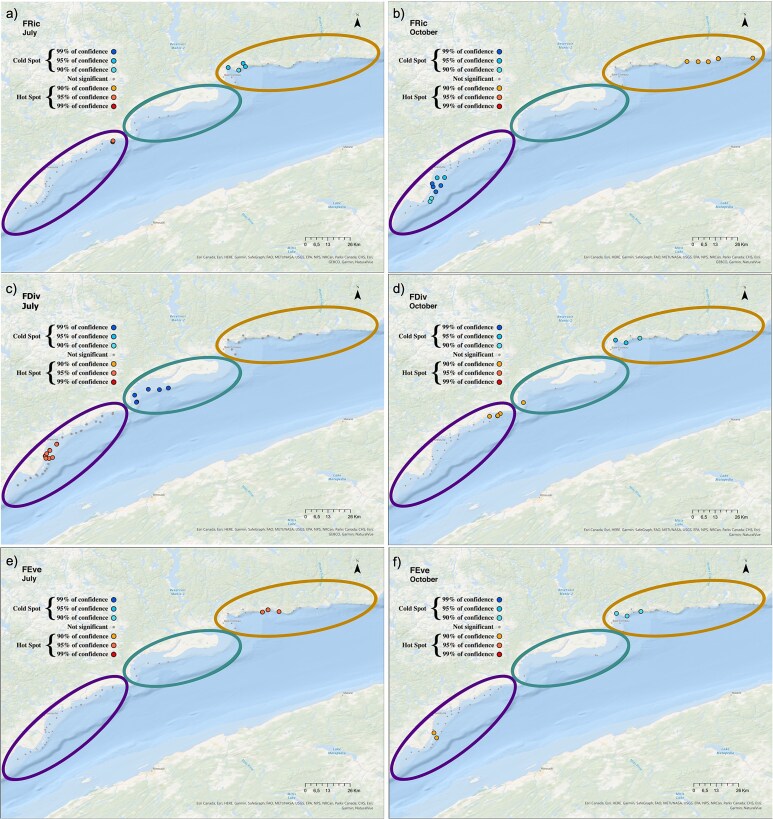
Maps indicating the distribution of the hotspots (red shades) and coldspots (blue shades) of (**a, b**) FRic, (**c, d**) FDiv and (**e, f**) FEve in July (left panels) and October (right panels), all years pooled. Sectors are represented by ellipsoids, “Forestville” in purple (left), “Manicouagan” in green (middle) and “B-C” in orange (right).

The spatial distribution of the functional biodiversity hotspots and coldspots varied between the 2 months and among indices (FRic, FDiv, FEve) along the coastal zone. FRic hotspots were located in Forestville and the coldspots in B-Cin July and showed an opposite pattern in October (Fig. 10a and b). FDiv hotspots were identified in Forestville and the coldspots in Manicouagan in July. Hotspots of FEve were detected in B-Cin July (Fig. 10c and e). In contrast, in October, hotspots of FDiv and FEve were found in Forestville (Fig. 10d and f), whereas coldspots occurred in B-C.

#### Trait-environment interactions

Functional diversity was influenced by different environmental factors in different ways (Table S12). Temperature had a positive effect on FRic within the range of 7.4–17.7°C, with an optimal point at 12.12°C and a negative effect below and above this range (Fig. 8d). FRic was also positively correlated with Chl-*a* concentration (Table S12). FDiv was positively influenced by temperature and Chl *a*, whereas FEve was positively influenced by salinity (Table S12). Predictability of the correlations was reliable for FRic and FDiv (deviance explained: 37.7% and 34.9%, respectively) but not for FEve (deviance explained 10%) (Table S12).

## DISCUSSION

The present study is the first to analyse the shallow coastal zooplankton in the LSLE at depth of <35 m, highlighting the driving role of seasonally and spatially variable environmental conditions in shaping community structure and biodiversity. In the following, we will discuss our results also in comparison with previous studies and the Atlantic Zone Monitoring Program, which focused on deeper pelagic areas (Laurentian Channel) of the LSLE (e.g. Runge and Simard, 1990; Plourde *et al*., 2002; Blais *et al*., 2021).

### Spatial and temporal variability in environmental conditions

In the LSLE, the shallow coastal zone (<35 m), similar to the deep zone of LSLE (>50 m), is characterized by strong seasonality of environmental conditions, which is highly dependent on the condition of ice-cover during winter (Blais *et al*., 2018; Galbraith *et al*., 2019). However, higher temperatures occur compared to the Laurentian Channel in summer, generally related to the shallow depth of coastal environments, strongly depending on air temperatures (Galbraith *et al*., 2019) and wind-related surface currents (El-Sabh, 1979). The mean temperatures recorded in July and October 2019 to 2021 in the present study are in accordance with temperatures recorded by thermographs in B-C in the same years (Galbraith *et al*., 2020, 2021, 2022), showing seasonal interannual variations. The temperature in 2019 was characterized by moderate and strong positive anomalies in July and October, respectively, compared to a 30-year mean (Galbraith *et al*., 2020). In 2020, July showed a strong positive anomaly, whereas October was colder than normal (Galbraith *et al*., 2021). In 2021, strong and very strong positive anomalies occurred in July and October (Galbraith *et al*., 2022). This illustrates the warmest July occurred in 2020 and the warmest October in 2021. As temperature directly acts on the physiology of zooplankton, it is one of the main factors influencing species distribution and composition in the present study, as will be discussed below.

Coastal waters were less salty in July than in October due to the freshet, especially in the sectors of Manicouagan and Forestville, where river runoffs of several tributaries bring more freshwater into the coastal zone in spring and early summer, as it has also been observed along the northern coast of the GSL where the Romaine River flows (Senneville *et al*., 2018). Therefore, these rivers can indirectly influence coastal zooplankton communities and could help explain differences between zooplankton composition, abundance and diversities among sectors. Nutrient and Chl-*a* concentrations were generally higher in October compared to July, potentially reflecting seasonal nutrient upwelling induced by storms or by high tidal amplitude in autumn (Blais *et al*., 2018). This nutrient availability can promote algal growth in the coastal zone. These results differ from those obtained from long-term Chl *a* climatology in the LSLE, showing the peak of the phytoplankton bloom in June/July with a decrease toward October (Blais *et al*., 2023; Laliberté and Larouche, 2023). This shows that annual medium-scale variability in coastal zones can be different from low spatial resolution long-term trends of the entire LSLE. Sector-specific environmental differentiation was more pronounced in summer than in autumn, showing highest temperatures, especially in July, in the Manicouagan sector, whereas Forestville was the coldest sector and B-C the saltiest. This might be the result of geographic locations and differences in bathymetry. For example, in the shallow bay of the Manicouagan sector the water may experience more intense solar heating over the tidal cycle than in the Forestville sector, which consists of an elongated coastal stretch, connected to colder surface currents originating from the upwelling at the head of the LSLE (Vézina *et al*., 1995). B-C, being the most saline station, might be influenced by warmer and more saline water entering from the GSL (El-Sabh, 1976).

### Environmental influence on the variability of zooplankton communities

Temperature, salinity and Chl *a* are the factors that mostly explain the variability of the zooplankton communities in the coastal zone of LSLE. Zooplankton species are poikilotherms and thus highly dependent on the temperature of their environment (Castellani and Edwards, 2017), which modulates key biological processes, such as growth and reproduction. Higher water temperature coincided with the occurrence of meroplankton, which was more abundant in July than in October, matching the reproduction timing of benthic species, as in the GSL, the Arctic and the Baltic Sea (Aucoin *et al*., 2004; Schulz *et al*., 2012; Deblois *et al*., 2022; Søreide *et al*., 2022). In July 2020, these higher temperatures induced a higher proportion of meroplankton, mainly composed of Bivalvia veliger, compared to other years. Meroplankton is favored by rising surface temperatures, increasing larval survival (Strand *et al*., 2020) as seen for some bivalve species in the North Sea (Kirby *et al*., 2008). Likewise, in GSL, around the Magdalen Islands, *Mytilus edulis* reproduction peaks in June and July (Aucoin *et al*., 2004). Within the same area, Guillou *et al*. (2023) showed that the temperature had a positive effect on this bivalve species reproduction up to a threshold of 20°C; that was, however, not reached in the present study. As we sampled only one week in July each year, we could have missed the reproduction peak in 2019 and 2021, potentially explaining why meroplankton was found in lower proportions than in 2020. The timing of meroplankton occurrence in the present study is in accordance with Harvey and Devine (2009), who found a higher abundance of meroplankton and especially of Bivalvia, in June than in November in the middle of the LSLE. However, abundances of meroplankton are more than three times higher in the coastal zone (present study) than in the deeper habitats of the LSLE in June 2007 (Harvey and Devine, 2009), owing to the presence of a more abundant benthic community in the coastal area (Archambault and Bourget, 1999; Titelman and Fiksen, 2004; Le Corre *et al*., 2015; Pineda-Metz and Montiel, 2021). Furthermore, differences in abundances between coastal and deep-water habitats might be explained by the hydrodynamics of the LSLE, including wind, which is as an important factor in the dispersion or aggregation of meroplankton in coastal areas as shown by Senneville *et al*. (2018).

Different studies in estuarine environments showed salinity as the principal factor driving zooplankton communities (Helenius *et al*., 2017; Labuce *et al*., 2021). However, in the present study, there was no evidence that salinity affected total abundance and diversities, but it had an influence on species composition, especially in October (Fig. 7). Salinity tolerance might vary among different taxa, resulting in species-specific distribution patterns (Runge and Simard, 1990; Laprise and Dodson, 1994). As we did not cover the entire salinity gradient of the SLE from zero to marine conditions, freshwater and oligohaline indicator species, such as *Daphnia* sp. and *Bosmina* spp., respectively, occurring only once at one station, could not explain the salinity effect. Two species of Copepoda, though, showed differences in distribution pattern according to salinity, *i.e*. *A. longiremis* and *O. similis,* which were the most abundant species in our study regardless of sector, month or year (Table II). Indeed, *A. longiremis*, a true euryhaline species that tolerates low salinities but avoids warm surface layers (Schulz *et al*., 2012), was more abundant in the Forestville than the B-C sector, the former being characterized by lower mean salinity and lower temperature. In contrast, *O. similis* has low tolerance to low temperatures and low salinities (Ward and Hirst, 2007; Teodósio and Barbosa, 2021), favoring temperatures between 15 and 20°C (Castellani *et al*., 2016). Highest reproductive success was found in salinities of ~30 PSU (Poulet and Harel, 1983), as in the B-C sector. Thus, spatial distribution might be linked to salinity for *O. similis* and to temperature for *A. longiremis*. Unfortunately, the lack of ecophysiological tolerance data for most species in the community prevented us from incorporating this functional trait into our analyses. However, future work could benefit from including such data, as it has been shown to be crucial for understanding zooplankton distribution.

**Table II:** Taxa contribution (%) to species composition based on SIMPER (PRIMER®). The five first taxa in the table mainly contributed to species composition.

Species	July.2019	July.2020	July.2021	October.2019	October.2020	October.2021
*Acartia longiremis*	10	7.1	16.7	20.1	19.4	
Bivalvia	15.8	24.9	9.1			
*Fritillaria* sp.	19.7	4.8	16	31.3	24.1	
*Oithona similis*	11.9	14,4		8.8	18.8	18.6
Cirripedia	6.2	5.5	7.8	4.2	4.6	
*Pseudocalanus* spp.		5.9				14.3
*Evadne* sp.			7.2			6.5
Euphausiidae			6.9	6.7	6.2	
Echinodermata	7.1					
*Eurytemora herdmani*		5.6				
*Centropages hamatus*						11
*Temora longicornis*						17.3
*Calanus finmarchicus*		5	6.6			
*Tortanus discaudatus*						6.5
Total contribution	70.7	73.2	70.3	71.1	73.1	74.2

Total zooplankton abundance was positively correlated to Chl *a* (GAMs), which indicates availability of autotrophic phytoplankton prey that promotes individual survival and hatching (Dufour *et al*., 2010; Dudeck *et al*., 2021). However, regarding the functional traits of trophic type, the proportion of herbivorous was higher in July than in October. In 2019 and 2020, low Chl *a* concentration in July coincided with high proportion of herbivorous and omnivorous, suggesting high grazing pressure by abundant zooplankton. Thus, we might have sampled during the part of the plankton succession when phytoplankton concentration was consumed and zooplankton abundance was still high (Winder and Varpe, 2020). Alternatively, lower Chl *a* concentrations in July compared to October might be due to the sampling time in July, missing the first large bloom that appeared, for instance, already in April in the coastal zone of the North Shore of the GSL (Deblois *et al*., 2022). In October 2019 and 2020, the proportion of omnivorous was mainly composed of Appendicularia, which might be linked to the phytoplankton bloom in October due to their capacity to ingest smaller phytoplankton and particles than other mesozooplankton organisms (Fernández *et al*., 2004).

### Zooplankton composition compared to deeper habitats

Zooplankton species in the coastal zone of the LSLE are similar to the ones found in deeper habitats (35–300 m depth) of the LSLE in previous studies (Plourde *et al*., 2002; Harvey and Devine, 2009; Blais *et al*., 2018), but differ in proportions, abundances and timing of occurrence, apart the common dominance of Copepoda. The small cyclopoid *O. similis* is one of the most abundant species in the LSLE, where its abundance peaks in July in shallow waters (present study) in contrast to autumn in deeper habitats (Plourde *et al*., 2002; Savenkoff *et al*., 2017). Furthermore, large copepods, such as *Calanus finmarchicus* and *Calanus hyperboreus* are much less abundant in the coastal zone than in the deeper habitats of the LSLE (Plourde *et al*., 2002; Harvey and Devine, 2009; Blais *et al*., 2018). Plourde *et al*. (2002) argue that small species, such as *Oithona* spp. and *Acartia* spp. are surface species, whereas bigger species, such as *Calanus* spp. remain in deeper layers to avoid predators during the day, showing diurnal vertical migration. Then, small species are more susceptible to being advected with surface currents than *Calanus* spp. and might aggregate in coastal retention areas (Savenkoff *et al*., 2017). This might explain the higher abundance of small copepods species and the lower abundance of *Calanus* spp. in the shallow coastal zone compared to the deeper LSLE. Zooplankton distribution is directly influenced by wind and tides, which are the major components in coastal areas that influence the surface layer (Archambault *et al*., 1998; Le Corre *et al*., 2015; Campos *et al*., 2017; Senneville *et al*., 2018) in contrast to the deeper zone. However, these physical factors, which could not be measured in our study, should be taken into consideration for future studies in order to better define the dispersal and origin of zooplankton and meroplankton in particular.

### Zooplankton taxonomic and functional diversities

Along the coastal zone, differences in spatial distribution patterns of hotspots and coldspots in both taxonomic and functional diversities are revealed, varying by sector and month in response to changing environmental conditions. Temperature explained most of the variance in species richness (18%), while the combination of temperature and Chl *a* were the best predictors of FRic, explaining 38% of the variance. Pomerleau *et al*. (2015) argued that abiotic factors could play a role in the expression of taxonomic and FRic, either through direct effects on species or indirect effects via responses reflected in functional traits. Only a low number of stations expressing hotspots or coldspots of FEve were found, and only 10% of the variance could be explained by environmental variables such as salinity and Chl *a.* The few occasions when FRic was low corresponded to low resource availability, suggesting that species favor complementary resource use instead of resource partitioning. High FEve was related to species using complementary resources found on a tropical-polar latitudinal gradient (Becker *et al*., 2021), then the seasonal and spatial variations of these resources would modulate the location of FEve hotspots. In contrast, the hotspots of Shannon-Wiener, Pielou evenness and FDiv occurred in the same sector in both months, therefore showing some seasonal stability. This could be related to a buffering effect on variability of environmental conditions due to the calculation including the number of species and the relative abundance in the Shannon-Wiener and Pielou evenness and for FDiv, the abundance-weighted functional differences among species (Mason *et al*., 2008).

Comparative indices of taxonomic and functional diversities showed a difference in their distribution of hotspots and coldspots. In the two months investigated, consistently, hotspots of the Shannon-Wiener index occurred in B-C while hotspots of FDiv were found in Forestville. As FDiv relates to how species abundances are distributed in functional trait space (Villéger *et al*., 2008), hotspots in the sector of Forestville indicate that few but highly abundant species (coldspots for Shannon-Wiener and Pielou evenness) showed a wide range of functional traits. Therefore, hotspots in the Forestville sector are likely to have a high degree of niche differentiation and thus might show low resource competition with more efficient use of resources by zooplankton communities (Mason *et al*., 2005). Overall, despite the low Shannon-Wiener index and due to high FDiv, the Forestville sector would still be expected to be resistant to disturbance or perturbation. Indeed, marine ecosystem stability could be promoted either by high taxonomic biodiversity, as seen in benthic ecosystems (Beaugrand, 2014) and/or by a high number of different functions, as found in coral reef ecosystems (Monaco and Prouzet, 2015). In contrast, when hotspots of Shannon-Wiener and Pilou evenness coincide with coldspots of FDiv, the community might be composed of a high number of species that show functional redundancy, however, they may also exhibit niche partitioning, which is not captured by the functional traits chosen, such as food preferences and environmental tolerance.

## CONCLUSION

Our study provides the first baseline assessment of the biodiversity of coastal zooplankton communities of the LSLE using complementary taxonomic and functional approaches. This dual approach enables us to gain a more integrative and holistic understanding of the communities under investigation. Total abundances were generally higher in July than in October in both 2019 and 2020; however, in 2021, we observed a shift toward higher abundances in both months. Furthermore, the mismatches observed in the spatial distribution of taxonomic and functional diversity hotspots—with taxonomic diversity (Shannon–Wiener index) concentrated in the B-C sector and FDiv in the Forestville sector -, highlight that an area may not appear taxonomically “diverse,” yet its functional diversity may be significant. This underscores the importance of considering such functionally diverse areas in conservation and management plans, even if they are not taxonomically rich. This baseline information will support future assessments of ecosystem change and management efforts in this area.

## Supplementary Material

Supplementary_material_SANTO_fbae073

## Data Availability

The data are archived at Observatoire global du Saint-Laurent (OGSL). Winkler, G., & Santo, M. (2022). Zooplancton de la zone côtière du nord de l’estuaire maritime du Saint-Laurent [Data set]. https://doi.org/10.26071/ogsl-3e9c3887-d969.
