# Supplementary material for: Assessing coastal zooplankton in the St. Lawrence estuary: spatio-temporal patterns of taxonomic and functional biodiversity
Source: J Plankton Res. 2025 Jan 30;47(1):fbae073. doi: 10.1093/plankt/fbae073 (PMC11781820; doi:10.1093/plankt/fbae073)
Supplement: Supplementary_material_SANTO_fbae073 [file supplementary_material_santo_fbae073.docx]

# Supplementary materiAL

**Table S1.** Summary of the information for the sampling campaigns. Year, month, number of sampling stations for environmental parameter (EP) and number of sampling stations for zooplankton (ZP) in bracket, vessel used, and environmental data available (D: depth, T: temperature, S: salinity, F: fluorescence, Chl*a*: chlorophyll *a*, pH: pH, Turb: turbidity, O2: oxygen, NO2+NO3: NO2-+NO3-, PO4: PO43-) are provided.

| **Year** | **Month** | **Nb of station**  **EP (ZP)** | **Vessel** | **EP data** |
| --- | --- | --- | --- | --- |
| 2019 | July | 6 (6) | CCGS LEIM | D, T, S, F, Chl*a*, O_2_ |
| 2019 | October | 8 (8) | CCGS LEIM | D, T, S, F, Chl*a* pH, Turb, NO_2_+NO_3_, PO_4_ |
| 2019 | October | 29 (29) | MACOMA | D, T, S, F, Chl*a*, pH, Turb |
| 2020 | July | 32 (32) | MACOMA | D, T, S, F, Chl*a*, pH, Turb, O_2_, NO_2_+NO_3_, PO_4_ |
| 2020 | October | 10 (10) | CCGS LEIM | D, T, S, F, Chl*a*, pH, Turb, O_2_, NO_2_+NO_3_, PO_4_ |
| 2021 | July | 34 (34) | MACOMA | D, T, S, F, Chl*a*, pH, Turb, O_2_, NO_2_+NO_3_, PO_4_ |
| 2021 | October | 4 (9) | CCGS PERLEY | D, T, S, F, Chl*a*, Turb, O_2_, NO_2_+NO_3_, PO_4_ |

**Table S2.** List of the taxa identified with information on phylogenetic, life stages and associated functional traits.

**Table S2 (continued).**

[1] Benedetti, F., Gasparini, S. and Ayata, S. D. (2016). Identifying copepod functional groups from species functional traits. *Journal of Plankton Research*, **38**(1), 159-166.

[2] Castellani, C. and Edwards, M. (Eds.). (2017). Marine Plankton: A practical guide to ecology, methodology, and taxonomy. *Oxford University Press*, 704 p.

[3] Venello, T. A., Sastri, A. R., Galbraith, M. D. and Dower, J. F. (2021). Zooplankton functional group responses to environmental drivers off the west coast of Vancouver Island, Canada. *Progress in Oceanography*, 190, 102482.

[4] Johnson, W. S., and Allen, D. M. (2012). Zooplankton of the Atlantic and Gulf coasts: a guide to their identification and ecology. *JHU Press*.

[5] Murphy, J. A. and Cohen, R. E. (1978). A guide to the developmental stages of common coastal, Georges Bank and Gulf of Maine copepods. National Marine Fisheries Service, Northeast Fisheries Center, Woods Hole Laboratory Reference Co. No. NOAA NEFSC.78-53, 56 pp.

[6] Sars, G. O. (1898). On the propagation and early development of Euphausiidae (No. 11). *Cammermeyers*.

[7] Akther, H., Agersted, M. D. and Olesen, J. (2015) Naupliar and Metanaupliar Development of *Thysanoessa raschii* (Malacostraca, Euphausiacea) from Godthåbsfjord, Greenland, with a Reinstatement of the Ancestral Status of the Free-Living Nauplius in Malacostracan Evolution. *PLOS ONE* **10**(12): e0141955.

[8] Blachowiak-Samolyk, K., Kwasniewski, S., Dmoch, K., Hop, H., and Falk-Petersen, S. (2007). Trophic structure of zooplankton in the Fram Strait in spring and autumn 2003. *Deep Sea Research Part II: Topical Studies in Oceanography*, **54**(23-26), 2716-2728.

[9] http://www.marinespecies.org/

[10] González, A. R., Guerra-García, J. M., Maestre, M. J., Ruiz-Tabares, A., Espinosa, F., Gordillo, I., Sánchez-Moyano, J. E. and García-Gómez, J. C. (2008). Community structure of caprellids (Crustacea: Amphipoda: Caprellidae) on seagrasses from southern Spain. *Helgoland Marine Research*, **62**, 189-199.

[11] Kjellerup, S. and Kiørboe, T. (2012). Prey detection in a cruising copepod. *Biology letters*, **8**(3), 438-441.

[12] Pomerleau, C., Sastri, A. R. and Beisner, B. E. (2015). Evaluation of functional trait diversity for marine zooplankton communities in the Northeast subarctic Pacific Ocean. *Journal of Plankton Research*, **37**(4), 712-726.

[13] Mullin, M. M. (1979). Differential predation by the carnivorous marine copepod, *Tortanus discaudatus*. *Limnology and Oceanography*, **24**(4), 774-777.

[14] Strathmann, R. R., Jahn, T. L. and Fonseca, J. R. (1972). Suspension feeding by marine invertebrate larvae: clearance of particles by ciliated bands of a rotifer, pluteus, and trochophore. *The Biological Bulletin*, **142**(3), 505-519.

[15] Vogt, R. J., Peres‐Neto, P. R. and Beisner, B. E. (2013). Using functional traits to investigate the determinants of crustacean zooplankton community structure. *Oikos*, **122**(12), 1700-1709.

[16] Kiørboe, T. (2011). How zooplankton feed: mechanisms, traits and trade‐offs. *Biological reviews*, **86**(2), 311-339.

[17] Caine, E. A. (1974). Comparative functional morphology of feeding in three species of caprellids (Crustacea, Amphipoda) from the northwestern Florida Gulf Coast. *Journal of Experimental Marine Biology and Ecology,* **15**(1), 81-96.

[18] Lindegren, M., Thomas, M. K., Jónasdóttir, S. H., Nielsen, T. G. and Munk, P. (2020). Environmental niche separation promotes coexistence among ecologically similar zooplankton species—North Sea copepods as a case study. *Limnology and Oceanography*, **65**(3), 545-556.

[19] Barton, A. D., Pershing, A. J., Litchman, E., Record, N. R., Edwards, K. F., Finkel, Z. V., Kiørboe, T. and Ward, B. A. (2013). The biogeography of marine plankton traits. *Ecology letters*, **16**(4), 522-534.

[20] Brun, P., Payne, M. R. and Kiørboe, T. (2017). A trait database for marine copepods. *Earth System Science Data*, **9**(1), 99-113.

[21] Moller, T. H. (1978). Feeding behaviour of larvae and postlarvae of *Macrobrachium rosenbergii* (De Man)(Crustacea: Palaemonidae). *Journal of Experimental Marine Biology and Ecology***, 35**(3), 251-258.

[22] Laboratory observations

[23] WoRMS Editorial Board (2023). World Register of Marine Species. Available from https://www.marinespecies.org at VLIZ. Accessed 2023-04-04. doi:10.14284/170

[1] Benedetti, F., Gasparini, S., & Ayata, S. D. (2016). Identifying copepod functional groups from species functional traits. Journal of Plankton Research, 38(1), 159-166. doi: 10.1093/plankt/fbv096. Epub 2015 Nov 3. PMID: 26811565; PMCID: PMC4722884.

[2] Castellani, C., & Edwards, M. (Eds.). (2017). Marine Plankton: A practical guide to ecology, methodology, and taxonomy. Oxford University Press.

[3] Venello, T. A., Sastri, A. R., Galbraith, M. D., & Dower, J. F. (2021). Zooplankton functional group responses to environmental drivers off the west coast of Vancouver Island, Canada. Progress in Oceanography, 190, 102482.

[4] Johnson, W. S., & Allen, D. M. (2012). Zooplankton of the Atlantic and Gulf coasts: a guide to their identification and ecology. JHU Press.

[5] Murphy, J.A. & R.E. Cohen. (1978). A guide to the developmental stages of common coastal, Georges Bank and Gulf of Maine copepods. National Marine Fisheries Service, Northeast Fisheries Center, Woods Hole Laboratory Reference Co. No. NOAA NEFSC.78-53, 56 pp.

[6] Sars, G. O. (1898). On the propagation and early development of Euphausiidae (No. 11). Cammermeyers.

[7] Akther H, Agersted MD, Olesen J (2015) Naupliar and Metanaupliar Development of Thysanoessa raschii (Malacostraca, Euphausiacea) from Godthåbsfjord, Greenland, with a Reinstatement of the Ancestral Status of the Free-Living Nauplius in Malacostracan Evolution. PLOS ONE 10(12): e0141955.

[8] Blachowiak-Samolyk, K., Kwasniewski, S., Dmoch, K., Hop, H., & Falk-Petersen, S. (2007). Trophic structure of zooplankton in the Fram Strait in spring and autumn 2003. Deep Sea Research Part II: Topical Studies in Oceanography, 54(23-26), 2716-2728.

[9] http://www.marinespecies.org/

[10] González, A. R., Guerra-García, J. M., Maestre, M. J., Ruiz-Tabares, A., Espinosa, F., Gordillo, I., ... & García-Gómez, J. C. (2008). Community structure of caprellids (Crustacea: Amphipoda: Caprellidae) on seagrasses from southern Spain. Helgoland Marine Research, 62, 189-199.

[11] Kjellerup, S., & Kiørboe, T. (2012). Prey detection in a cruising copepod. Biology letters, 8(3), 438-441.

[12] Pomerleau, C., Sastri, A. R., & Beisner, B. E. (2015). Evaluation of functional trait diversity for marine zooplankton communities in the Northeast subarctic Pacific Ocean. Journal of Plankton Research, 37(4), 712-726.

[13] Mullin, M. M. (1979). Differential predation by the carnivorous marine copepod, Tortanus discaudatus 1. Limnology and Oceanography, 24(4), 774-777.

[14] Strathmann, R. R., Jahn, T. L., & Fonseca, J. R. (1972). Suspension feeding by marine invertebrate larvae: clearance of particles by ciliated bands of a rotifer, pluteus, and trochophore. The Biological Bulletin, 142(3), 505-519.

[15] Vogt, R. J., Peres‐Neto, P. R., & Beisner, B. E. (2013). Using functional traits to investigate the determinants of crustacean zooplankton community structure. Oikos, 122(12), 1700-1709.

[16] Kiørboe, T. (2011). How zooplankton feed: mechanisms, traits and trade‐offs. Biological reviews, 86(2), 311-339.

[17] Caine, E. A. (1974). Comparative functional morphology of feeding in three species of caprellids (Crustacea, Amphipoda) from the northwestern Florida Gulf Coast. Journal of Experimental Marine Biology and Ecology, 15(1), 81-96.

[18] Lindegren, M., Thomas, M. K., Jónasdóttir, S. H., Nielsen, T. G., & Munk, P. (2020). Environmental niche separation promotes coexistence among ecologically similar zooplankton species—North Sea copepods as a case study. Limnology and Oceanography, 65(3), 545-556.

[19] Barton, A. D., Pershing, A. J., Litchman, E., Record, N. R., Edwards, K. F., Finkel, Z. V., ... & Ward, B. A. (2013). The biogeography of marine plankton traits. Ecology letters, 16(4), 522-534.

[20] Brun, P., Payne, M. R., & Kiørboe, T. (2017). A trait database for marine copepods. Earth System Science Data, 9(1), 99-113.

[21] Moller, T. H. (1978). Feeding behaviour of larvae and postlarvae of Macrobrachium rosenbergii (De Man)(Crustacea: Palaemonidae). Journal of Experimental Marine Biology and Ecology, 35(3), 251-258.

[22] laboratory observations

[23] WoRMS Editorial Board (2023). World Register of Marine Species. Available from https://www.marinespecies.org at VLIZ. Accessed 2023-04-04. doi:10.14284/170

[1] Benedetti, F., Gasparini, S., & Ayata, S. D. (2016). Identifying copepod functional groups from species functional traits. Journal of Plankton Research, 38(1), 159-166. doi: 10.1093/plankt/fbv096. Epub 2015 Nov 3. PMID: 26811565; PMCID: PMC4722884.

[2] Castellani, C., & Edwards, M. (Eds.). (2017). Marine Plankton: A practical guide to ecology, methodology, and taxonomy. Oxford University Press.

[3] Venello, T. A., Sastri, A. R., Galbraith, M. D., & Dower, J. F. (2021). Zooplankton functional group responses to environmental drivers off the west coast of Vancouver Island, Canada. Progress in Oceanography, 190, 102482.

[4] Johnson, W. S., & Allen, D. M. (2012). Zooplankton of the Atlantic and Gulf coasts: a guide to their identification and ecology. JHU Press.

[5] Murphy, J.A. & R.E. Cohen. (1978). A guide to the developmental stages of common coastal, Georges Bank and Gulf of Maine copepods. National Marine Fisheries Service, Northeast Fisheries Center, Woods Hole Laboratory Reference Co. No. NOAA NEFSC.78-53, 56 pp.

[6] Sars, G. O. (1898). On the propagation and early development of Euphausiidae (No. 11). Cammermeyers.

[7] Akther H, Agersted MD, Olesen J (2015) Naupliar and Metanaupliar Development of Thysanoessa raschii (Malacostraca, Euphausiacea) from Godthåbsfjord, Greenland, with a Reinstatement of the Ancestral Status of the Free-Living Nauplius in Malacostracan Evolution. PLOS ONE 10(12): e0141955.

[8] Blachowiak-Samolyk, K., Kwasniewski, S., Dmoch, K., Hop, H., & Falk-Petersen, S. (2007). Trophic structure of zooplankton in the Fram Strait in spring and autumn 2003. Deep Sea Research Part II: Topical Studies in Oceanography, 54(23-26), 2716-2728.

[9] http://www.marinespecies.org/

[10] González, A. R., Guerra-García, J. M., Maestre, M. J., Ruiz-Tabares, A., Espinosa, F., Gordillo, I., ... & García-Gómez, J. C. (2008). Community structure of caprellids (Crustacea: Amphipoda: Caprellidae) on seagrasses from southern Spain. Helgoland Marine Research, 62, 189-199.

[11] Kjellerup, S., & Kiørboe, T. (2012). Prey detection in a cruising copepod. Biology letters, 8(3), 438-441.

[12] Pomerleau, C., Sastri, A. R., & Beisner, B. E. (2015). Evaluation of functional trait diversity for marine zooplankton communities in the Northeast subarctic Pacific Ocean. Journal of Plankton Research, 37(4), 712-726.

[13] Mullin, M. M. (1979). Differential predation by the carnivorous marine copepod, Tortanus discaudatus 1. Limnology and Oceanography, 24(4), 774-777.

[14] Strathmann, R. R., Jahn, T. L., & Fonseca, J. R. (1972). Suspension feeding by marine invertebrate larvae: clearance of particles by ciliated bands of a rotifer, pluteus, and trochophore. The Biological Bulletin, 142(3), 505-519.

[15] Vogt, R. J., Peres‐Neto, P. R., & Beisner, B. E. (2013). Using functional traits to investigate the determinants of crustacean zooplankton community structure. Oikos, 122(12), 1700-1709.

[16] Kiørboe, T. (2011). How zooplankton feed: mechanisms, traits and trade‐offs. Biological reviews, 86(2), 311-339.

[17] Caine, E. A. (1974). Comparative functional morphology of feeding in three species of caprellids (Crustacea, Amphipoda) from the northwestern Florida Gulf Coast. Journal of Experimental Marine Biology and Ecology, 15(1), 81-96.

[18] Lindegren, M., Thomas, M. K., Jónasdóttir, S. H., Nielsen, T. G., & Munk, P. (2020). Environmental niche separation promotes coexistence among ecologically similar zooplankton species—North Sea copepods as a case study. Limnology and Oceanography, 65(3), 545-556.

[19] Barton, A. D., Pershing, A. J., Litchman, E., Record, N. R., Edwards, K. F., Finkel, Z. V., ... & Ward, B. A. (2013). The biogeography of marine plankton traits. Ecology letters, 16(4), 522-534.

[20] Brun, P., Payne, M. R., & Kiørboe, T. (2017). A trait database for marine copepods. Earth System Science Data, 9(1), 99-113.

[21] Moller, T. H. (1978). Feeding behaviour of larvae and postlarvae of Macrobrachium rosenbergii (De Man)(Crustacea: Palaemonidae). Journal of Experimental Marine Biology and Ecology, 35(3), 251-258.

[22] laboratory observations

[23] WoRMS Editorial Board (2023). World Register of Marine Species. Available from https://www.marinespecies.org at VLIZ. Accessed 2023-04-04. doi:10.14284/170

[1] Benedetti, F., Gasparini, S., & Ayata, S. D. (2016). Identifying copepod functional groups from species functional traits. Journal of Plankton Research, 38(1), 159-166. doi: 10.1093/plankt/fbv096. Epub 2015 Nov 3. PMID: 26811565; PMCID: PMC4722884.

[2] Castellani, C., & Edwards, M. (Eds.). (2017). Marine Plankton: A practical guide to ecology, methodology, and taxonomy. Oxford University Press.

[3] Venello, T. A., Sastri, A. R., Galbraith, M. D., & Dower, J. F. (2021). Zooplankton functional group responses to environmental drivers off the west coast of Vancouver Island, Canada. Progress in Oceanography, 190, 102482.

[4] Johnson, W. S., & Allen, D. M. (2012). Zooplankton of the Atlantic and Gulf coasts: a guide to their identification and ecology. JHU Press.

[5] Murphy, J.A. & R.E. Cohen. (1978). A guide to the developmental stages of common coastal, Georges Bank and Gulf of Maine copepods. National Marine Fisheries Service, Northeast Fisheries Center, Woods Hole Laboratory Reference Co. No. NOAA NEFSC.78-53, 56 pp.

[6] Sars, G. O. (1898). On the propagation and early development of Euphausiidae (No. 11). Cammermeyers.

[7] Akther H, Agersted MD, Olesen J (2015) Naupliar and Metanaupliar Development of Thysanoessa raschii (Malacostraca, Euphausiacea) from Godthåbsfjord, Greenland, with a Reinstatement of the Ancestral Status of the Free-Living Nauplius in Malacostracan Evolution. PLOS ONE 10(12): e0141955.

[8] Blachowiak-Samolyk, K., Kwasniewski, S., Dmoch, K., Hop, H., & Falk-Petersen, S. (2007). Trophic structure of zooplankton in the Fram Strait in spring and autumn 2003. Deep Sea Research Part II: Topical Studies in Oceanography, 54(23-26), 2716-2728.

[9] http://www.marinespecies.org/

[10] González, A. R., Guerra-García, J. M., Maestre, M. J., Ruiz-Tabares, A., Espinosa, F., Gordillo, I., ... & García-Gómez, J. C. (2008). Community structure of caprellids (Crustacea: Amphipoda: Caprellidae) on seagrasses from southern Spain. Helgoland Marine Research, 62, 189-199.

[11] Kjellerup, S., & Kiørboe, T. (2012). Prey detection in a cruising copepod. Biology letters, 8(3), 438-441.

[12] Pomerleau, C., Sastri, A. R., & Beisner, B. E. (2015). Evaluation of functional trait diversity for marine zooplankton communities in the Northeast subarctic Pacific Ocean. Journal of Plankton Research, 37(4), 712-726.

[13] Mullin, M. M. (1979). Differential predation by the carnivorous marine copepod, Tortanus discaudatus 1. Limnology and Oceanography, 24(4), 774-777.

[14] Strathmann, R. R., Jahn, T. L., & Fonseca, J. R. (1972). Suspension feeding by marine invertebrate larvae: clearance of particles by ciliated bands of a rotifer, pluteus, and trochophore. The Biological Bulletin, 142(3), 505-519.

[15] Vogt, R. J., Peres‐Neto, P. R., & Beisner, B. E. (2013). Using functional traits to investigate the determinants of crustacean zooplankton community structure. Oikos, 122(12), 1700-1709.

[16] Kiørboe, T. (2011). How zooplankton feed: mechanisms, traits and trade‐offs. Biological reviews, 86(2), 311-339.

[17] Caine, E. A. (1974). Comparative functional morphology of feeding in three species of caprellids (Crustacea, Amphipoda) from the northwestern Florida Gulf Coast. Journal of Experimental Marine Biology and Ecology, 15(1), 81-96.

[18] Lindegren, M., Thomas, M. K., Jónasdóttir, S. H., Nielsen, T. G., & Munk, P. (2020). Environmental niche separation promotes coexistence among ecologically similar zooplankton species—North Sea copepods as a case study. Limnology and Oceanography, 65(3), 545-556.

[19] Barton, A. D., Pershing, A. J., Litchman, E., Record, N. R., Edwards, K. F., Finkel, Z. V., ... & Ward, B. A. (2013). The biogeography of marine plankton traits. Ecology letters, 16(4), 522-534.

[20] Brun, P., Payne, M. R., & Kiørboe, T. (2017). A trait database for marine copepods. Earth System Science Data, 9(1), 99-113.

[21] Moller, T. H. (1978). Feeding behaviour of larvae and postlarvae of Macrobrachium rosenbergii (De Man)(Crustacea: Palaemonidae). Journal of Experimental Marine Biology and Ecology, 35(3), 251-258.

[22] laboratory observations

[23] WoRMS Editorial Board (2023). World Register of Marine Species. Available from https://www.marinespecies.org at VLIZ. Accessed 2023-04-04. doi:10.14284/170

**Table S3.** List of taxa with associated biomass formulas, expressed in mgC. Biomass data were calculated based on size (PL= prosome length, TL and L = total length, in mm) and specialized literature. Copepod species formulas were used for copepodite stages. Taxa with different specific stages are specified in brackets.

**Reference biomass table:**

Arendt, K. E., Juul-Pedersen, T., Mortensen, J., Blicher, M. E. and Rysgaard, S. (2013). A 5-year study of seasonal patterns in mesozooplankton community structure in a sub-Arctic fjord reveals dominance of *Microsetella norvegica* (Crustacea, Copepoda). *Journal of Plankton Research*, **35**(1), 105-120.

Burkill, P. H. and Kendall, T. F. (1982). Production of the copepod *Eurytemora affinis* in the Bristol Channel. *Marine Ecology Progress Series*, **7**(1), 21-31.

Cohen, R. E. and Lough, R. G. (1981). Length-weight relationships for several copepods dominant in the Georges Bank-Gulf of Maine area. *Journal of Northwest Atlantic Fishery Science*, 2.

Deibel, D. (1986). Feeding mechanism and house of the appendicularian *Oikopleura vanhoeffeni*. *Marine Biology*, **93**, 429-436.

Dumont, H. J., Van de Velde, I. and Dumont, S. (1975). The dry weight estimate of biomass in a selection of Cladocera, Copepoda and Rotifera from the plankton, periphyton and benthos of continental waters. *Oecologia*, **19**(1), 75-97.

Fotel, F. L., Jensen, N. J., Wittrup, L. and Hansen, B. W. (1999). In situ and laboratory growth by a population of blue mussel larvae (*Mytilus edulis L*.) from a Danish embayment, Knebel Vig. *Journal of Experimental Marine Biology and Ecology*, **233**(2), 213-230.

Hay, S. J., Kiørboe, T. and Matthews, A. (1991). Zooplankton biomass and production in the North Sea during the autumn circulation experiment, October 1987–March 1988. *Continental Shelf Researc*h, **11**(12), 1453-1476.

Hopcroft, R. R., Clarke, C., Nelson, R. J. and Raskoff, K. A. (2005). Zooplankton communities of the Arctic’s Canada Basin: the contribution by smaller taxa. *Polar Biology*, 28, 198-206.

Hopcroft, R. R., Kosobokova, K. N. and Pinchuk, A. I. (2010). Zooplankton community patterns in the Chukchi Sea during summer 2004. *Deep Sea Research Part II: Topical Studies in Oceanography*, **57**(1-2), 27-39.

Laurence, G. C. (1979). Larval length-weight relations for 7 species of northwest Atlantic fishes reared in the laboratory. *Fishery* *Bulletin*, **76**(4), 890-895.

Legendre, L. and Michaud, J. (1998). Flux of biogenic carbon in oceans: size-dependent regulation by pelagic food webs. *Marine* *Ecology Progress Series*, 164, 1-11.

Martins, I., Maranhão, P. and Marques, J. C. (2002). Modelling the effects of salinity variation on *Echinogammarus marinus* Leach (Amphipoda, Gammaridae) density and biomass in the Mondego estuary (Western Portugal). *Ecological Modelling*, **152**(2-3), 247-260.

Mason, C. F. and Abdul‐Hussein, M. M. (1991). Population dynamics and production of *Daphnia hyalina* and *Bosmina longirostris* in a shallow, eutrophic reservoir. *Freshwater Biology*, **25**(2), 243-260.

Mauchline, J. (1998).  The biology of calanoid copepods. *Advances in Marine Biol*ogy, Volume 33.

Moriarty, R. and O'brien, T. D. (2013). Distribution of mesozooplankton biomass in the global ocean. *Earth System Science* *Data*, **5**(1), 45-55.

Arendt, K. E., Juul-Pedersen, T., Mortensen, J., Blicher, M. E., & Rysgaard, S. (2013). A 5-year study of seasonal patterns in mesozooplankton community structure in a sub-Arctic fjord reveals dominance of *Microsetella norvegica* (Crustacea, Copepoda). Journal of plankton research, 35(1), 105-120.

Burkill, P. H., & Kendall, T. F. (1982). Production of the copepod *Eurytemora affinis* in the Bristol Channel. Marine Ecology Progress Series, 7(1), 21-31.

Cohen, R. E., & Lough, R. G. (1981). Length-weight relationships for several copepods dominant in the Georges Bank-Gulf of Maine area. Journal of Northwest Atlantic Fishery Science, 2.

Deibel, D. (1986). Feeding mechanism and house of the appendicularian *Oikopleura vanhoeffeni*. Marine Biology, 93, 429-436.

Dumont, H. J., Van de Velde, I., & Dumont, S. (1975). The dry weight estimate of biomass in a selection of Cladocera, Copepoda and Rotifera from the plankton, periphyton and benthos of continental waters. Oecologia, 19(1), 75-97.

Fotel, F. L., Jensen, N. J., Wittrup, L., & Hansen, B. W. (1999). In situ and laboratory growth by a population of blue mussel larvae (*Mytilus edulis* L.) from a Danish embayment, Knebel Vig. Journal of Experimental Marine Biology and Ecology, 233(2), 213-230.

Hay, S. J., Kiørboe, T., & Matthews, A. (1991). Zooplankton biomass and production in the North Sea during the autumn circulation experiment, October 1987–March 1988. Continental Shelf Research, 11(12), 1453-1476.

Hopcroft, R. R., Clarke, C., Nelson, R. J., & Raskoff, K. A. (2005). Zooplankton communities of the Arctic’s Canada Basin: the contribution by smaller taxa. Polar Biology, 28, 198-206.

Hopcroft, R. R., Kosobokova, K. N., & Pinchuk, A. I. (2010). Zooplankton community patterns in the Chukchi Sea during summer 2004. Deep Sea Research Part II: Topical Studies in Oceanography, 57(1-2), 27-39.

Laurence, G. C. (1979). Larval length-weight relations for 7 species of northwest Atlantic fishes reared in the laboratory. Fishery Bulletin, 76(4), 890-895.

Legendre, L., & Michaud, J. (1998). Flux of biogenic carbon in oceans: size-dependent regulation by pelagic food webs. Marine Ecology Progress Series, 164, 1-11.

Martins, I., Maranhão, P., & Marques, J. C. (2002). Modelling the effects of salinity variation on *Echinogammarus marinus* Leach (Amphipoda, Gammaridae) density and biomass in the Mondego estuary (Western Portugal). Ecological Modelling, 152(2-3), 247-260.

Mason, C. F., & Abdul‐Hussein, M. M. (1991). Population dynamics and production of *Daphnia hyalina* and *Bosmina longirostris* in a shallow, eutrophic reservoir. Freshwater Biology, 25(2), 243-260.

Mauchline, J. (1998). Adv. Mar. Biol. 33: The biology of calanoid copepods.

Moriarty, R., & O'brien, T. D. (2013). Distribution of mesozooplankton biomass in the global ocean. Earth System Science Data, 5(1), 45-55.

Pond, D. W., Tarling, G. A., Schmidt, K. and Everson, I. (2012). Diet and growth rates of *Meganyctiphanes norvegica* in autumn. *Marine Biology Research*, **8**(7), 615-623.

Robert, D., Castonguay, M. and Fortier, L. (2008). Effects of intra-and inter-annual variability in prey field on the feeding selectivity of larval Atlantic mackerel (*Scomber scombrus*). *Journal of Plankton Research*, **30**(6), 673-688.

Satapoomin, S. (1999). Carbon content of some common tropical Andaman Sea copepods. *Journal of Plankton Research*, **21**(11), 2117-2123.

Senneville, S., Schloss, I. R., Drouin, S. S. O., Bélanger, S., Winkler, G., Dumont, D., Johnston, P. and St-Onge, I. (2018). Moderate effect of damming the Romaine River (Quebec, Canada) on coastal plankton dynamics. *Estuarine, Coastal and Shelf Science*, 203, 29-43.

Teglhus, F. W., Agersted, M. D., Akther, H. and Nielsen, T. G. (2015). Distributions and seasonal abundances of krill eggs and larvae in the sub-Arctic Godthåbsfjord, SW Greenland. *Marine Ecology Progress Series*, 539, 111-125.

Thor, P., Nielsen, T. G., Tiselius, P., Juul-Pedersen, T., Michel, C., Møller, E. F., Dahl, K., Selander, E. and Gooding, S. (2005). Post-spring bloom community structure of pelagic copepods in the Disko Bay, Western Greenland. *Journal of Plankton* *Research*, **27**(4), 341-356.

Uye, S. I. (1982). Length-weight relationships of important zooplankton from the Inland Sea of Japan. *Journal of the* *Oceanographical Society of Japan*, 38, 149-158.

Uye, S. I., Aoto, I. and Onbé, T. (2002). Seasonal population dynamics and production *of Microsetella norvegica*, a widely distributed but little-studied marine planktonic harpacticoid copepod. *Journal of Plankton Research*, **24**(2), 143-153.

Walve, J. and Larsson, U. (1999). Carbon, nitrogen and phosphorus stoichiometry of crustacean zooplankton in the Baltic Sea: implications for nutrient recycling. *Journal of plankton research*, **21**(12), 2309-2321.

Wasson, K. M. and Watts, S. A. (2001). Reproductive endocrinology of sea urchins. *Developments in Aquaculture and Fisheries* *Science*, **32**, 43-57.

Pond, D. W., Tarling, G. A., Schmidt, K., & Everson, I. (2012). Diet and growth rates of *Meganyctiphanes norvegica* in autumn. Marine Biology Research, 8(7), 615-623.

Robert, D., Castonguay, M., & Fortier, L. (2008). Effects of intra-and inter-annual variability in prey field on the feeding selectivity of larval Atlantic mackerel (*Scomber scombrus*). Journal of Plankton Research, 30(6), 673-688.

Satapoomin, S. (1999). Carbon content of some common tropical Andaman Sea copepods. Journal of Plankton Research, 21(11), 2117-2123.

Senneville, S., Schloss, I. R., Drouin, S. S. O., Bélanger, S., Winkler, G., Dumont, D., Johnston, P., & St-Onge, I. (2018). Moderate effect of damming the Romaine River (Quebec, Canada) on coastal plankton dynamics. Estuarine, Coastal and Shelf Science, 203, 29-43.

Teglhus, F. W., Agersted, M. D., Akther, H., & Nielsen, T. G. (2015). Distributions and seasonal abundances of krill eggs and larvae in the sub-Arctic Godthåbsfjord, SW Greenland. Marine Ecology Progress Series, 539, 111-125.

Thor, P., Nielsen, T. G., Tiselius, P., Juul-Pedersen, T., Michel, C., Møller, E. F., Dahl, K., Selander, E., & Gooding, S. (2005). Post-spring bloom community structure of pelagic copepods in the Disko Bay, Western Greenland. Journal of Plankton Research, 27(4), 341-356.

Uye, S. I. (1982). Length-weight relationships of important zooplankton from the Inland Sea of Japan. Journal of the Oceanographical Society of Japan, 38, 149-158.

Uye, S. I., Aoto, I., & Onbé, T. (2002). Seasonal population dynamics and production *of Microsetella norvegica*, a widely distributed but little-studied marine planktonic harpacticoid copepod. Journal of Plankton Research, 24(2), 143-153.

Walve, J., & Larsson, U. (1999). Carbon, nitrogen and phosphorus stoichiometry of crustacean zooplankton in the Baltic Sea: implications for nutrient recycling. Journal of plankton research, 21(12), 2309-2321.

Wasson, K. M., & Watts, S. A. (2001). Reproductive endocrinology of sea urchins. In Developments in Aquaculture and Fisheries Science (Vol. 32, pp. 43-57). Elsevier.

**Table S4.** Summary of the GAM model selection for the biodiversity indices (species richness, Shannon-Wiener index, Pielou eveness, FRic: functional richness, FDiv: functional divergence, FEve: functional eveness) and total abundance interactions with environmental variables (temperature, salinity and Chl-*a*) for GAM analysis. The model with the best ∆AIC score (in bold) was used for the GAM analysis. If ∆AIC score was similar, the model with the fewest variables involved was selected, as the addition of a predictor did not increase the explanatory power of the model. The symbol “+“ indicates the selection of the smoothed variable for the model.

**Table S5.** Summary of the results of the permutational multivariate analysis of variance (PERMANOVA) testing for each environmental parameter as a function of the interaction of *Year*, *Month** and *Sector** and their 2- and 3- ways interactions.

R^2^

R^2^

R^2^

R^2^

R^2^

**PO_4_**

R^2^

**O_2_**

**NO_3_ / NO_2_**

**Table S6.** Summary of the results of permutational multivariate analysis of variance (PERMANOVA) indicating for significant differences of the interaction between the terms **Year*, **Month* and **Sector* and their interactions (*p < 0.05*) of environmental data and abundance. The presence of variable abbreviation in a given part indicates a significant difference. Significative differences between *Month* were represented above diagonal part of the table, and significant differences between *Month***Sector* in the below diagonal part of the table. Blue coloration highlights interactions among the three months of July, Green coloration among the three months of October and Orange coloration among sectors within a same month.

**Table S6 (continued).**

**Table S5 (continued)**

**Table S7.** Summary of the results of the permutational multivariate analysis of variance (PERMANOVA) testing for abundance, biomass, the five major species, taxonomic indices and functional indices as a function of the interaction of *Year**, *Month** and *Sector** and their 2- and 3-ways interactions.

R^2^

R^2^

R^2^

R^2^

**Table S7 (continued)**

R^2^

R^2^

R^2^

R^2^

**Table S7 (continued)**

R^2^

R^2^

R^2^

R^2^

R^2^

R^2^

R^2^

**Table S8.** Summary of PERMANOVA results of species assemblage based on Bray Curtis dissimilarity as a function of the interaction between the terms *Year*, *Month* and *Sector* and their interactions.

R^2^

**Table S9.** Summary of pairwise results (Bonferroni adjustment) of PERMANOVA of species composition (based on Bray-Curtis dissimilarity). Each X represented significative interactions among sectors, month and year. Orange coloration indicates interactions within the same month.

**Table S10.** Summary of pairwise results (Bonferroni adjustment) of PERMANOVA of species composition (based on Bray-Curtis dissimilarity) among month and years.

R^2^

**Table S11.** Summary of SIMPER dissimilarity matrix of species composition between sector, month and years. Orange cells represent dissimilarity within the same month. Dark cells show no data because represent the same sector within the same month. Grey parts are the data of the same sector between months within the same year.

**Table S12.** Summary of the results of generalized additive models (GAM) testing the effect of temperature, salinity and chl-a for abundance, species richness, Shannon-Wiener index, Pielou evenness, functional richness, functional divergence and functional evenness.

| **GAM** |  | Estimate | Std. Error | t value | Pr(>\|t\|) | edf | Ref.df | F | p-value | R-sq.(adj) | Deviance expl. (%) | REML | n |
| --- | --- | --- | --- | --- | --- | --- | --- | --- | --- | --- | --- | --- | --- |
| **Abundance** |  |  |  |  |  |  |  |  |  | 0.401 | 42.80 | 1227.6 | 123 |
|  | (Intercept) | 5540.1 | 474.1 | 11.69 | <2e-16 |  |  |  |  |  |  |  |  |
|  | s(Temperature) |  |  |  |  | 2.0159 | 9 | 1.315 | 0.0005 |  |  |  |  |
|  | s(Salinity) |  |  |  |  | 0.7652 | 9 | 0.14 | 0.1613 |  |  |  |  |
|  | s(Chla) |  |  |  |  | 2.7757 | 9 | 7.816 | < 2e-16 |  |  |  |  |
| **S** |  |  |  |  |  |  |  |  |  | 0.164 | 18.40 | 295.81 | 123 |
| Species |  |  |  |  |  |  |  |  |  |  |  |  |  |
| richness | (Intercept) | 18.024 | 0.2336 | 77.17 | <2e-16 |  |  |  |  |  |  |  |  |
|  | s(Temperature) |  |  |  |  | 3.017 | 9 | 2.655 | 2.57E-05 |  |  |  |  |
| **H'** |  |  |  |  |  |  |  |  |  | 0.139 | 16.80 | 64.673 | 123 |
| Shannon-Wiener | (Intercept) | 1.5658 | 0.03505 | 44.67 | <2e-16 |  |  |  |  |  |  |  |  |
| index | s(Temperature) |  |  |  |  | 2.1099 | 9 | 0.925 | 0.0071 |  |  |  |  |
|  | s(Salinity) |  |  |  |  | 0.5327 | 9 | 0.127 | 0.1328 |  |  |  |  |
|  | s(Chla) |  |  |  |  | 1.4798 | 9 | 0.957 | 0.0034 |  |  |  |  |
| **J'** |  |  |  |  |  |  |  |  |  | 0.106 | 12.60 | -75.41 | 123 |
| Pielou | (Intercept) | 0.5413 | 0.01129 | 47.97 | <2e-16 |  |  |  |  |  |  |  |  |
| evenness | s(Chla) |  |  |  |  | 1.576 | 9 | 1.119 | 0.0018 |  |  |  |  |
| index | s(Temperature) |  |  |  |  | 1.165 | 9 | 0.215 | 0.175 |  |  |  |  |
| **FRic** |  |  |  |  |  |  |  |  |  | 0.352 | 37.70 | -67.28 | 123 |
| Functional | (Intercept) | 0.5688 | 0.01175 | 48.4 | <2e-16 |  |  |  |  |  |  |  |  |
| richness | s(Temperature) |  |  |  |  | 2.981 | 9 | 6.882 | < 2e-16 |  |  |  |  |
|  | s(Chla) |  |  |  |  | 1.679 | 9 | 0.847 | 0.00791 |  |  |  |  |
| **FDiv** |  |  |  |  |  |  |  |  |  | 0.325 | 34.90 | -69.26 | 123 |
| Functional | (Intercept) | 0.7630 | 0.01164 | 65.53 | <2e-16 |  |  |  |  |  |  |  |  |
| divergence | s(Temperature) |  |  |  |  | 2.361 | 9 | 4.421 | <2e-16 |  |  |  |  |
|  | s(Chla) |  |  |  |  | 1.88 | 9 | 0.927 | 0.0083 |  |  |  |  |
| **FEve** |  |  |  |  |  |  |  |  |  | 0.0886 | 10.00 | -138.3 | 123 |
| Functional | (Intercept) | 0.3668 | 0.006795 | 53.98 | <2e-16 |  |  |  |  |  |  |  |  |
| evenness | s(Chla) |  |  |  |  | 0.6226 | 9 | 0.111 | 0.1994 |  |  |  |  |
|  | s(Salinity) |  |  |  |  | 0.908 | 9 | 1.092 | 0.0012 |  |  |  |  |

**Table S13.** Summary of PERMANOVA (with Monte-Carlo adjustment) results for the four different functional traits: life history (a), trophic type (b), feeding strategy (c) and size class (d), as a function of the interaction between the terms *Year*, *Month* and *Sector* and their interactions.

a)

a)

b)

b)

c)

c)

d)

d)
